# Meningitis in a French pig farmer caused by a serotype 2 *Streptococcus suis* isolate from the uncommon ST25 lineage

**DOI:** 10.1128/asmcr.00234-25

**Published:** 2026-05-12

**Authors:** M'hensa Vincent De Paul Bakpatina-Batako, Hubert Gantelet, Eric Lewandowski, Rodolphe Buzelé, Alain Delépine, Evelyne Gaillard, Sonia Lacouture, Kevin Li, Marcelo Gottschalk, Nahuel Fittipaldi

**Affiliations:** 1 Groupe de recherche sur les maladies infectieuses en production animale, and Centre de recherche en infectiologie porcine et avicole, Faculté de médecine vétérinaire, Université de Montréal 70354 , Saint-Hyacinthe, Quebec, Canada; 2 Ceva Biovac, Beaucouzé, France; 3 Centre Hospitalier Saint-Brieuc Paimpol Tréguier, Saint-Brieuc, France; 4 Selarl Vetarmor, Ploëzal, France; Pattern Bioscience, Austin, Texas, USA

**Keywords:** *Streptococcus suis*, sequence type 25 (ST25), zoonosis, meningitis, pig farmer, France, swine, phylogenetics, occupational exposure, emerging pathogen

## Abstract

**Background:**

Human *Streptococcus suis* infections occur worldwide but are most common in East and Southeast Asia. In Europe, most cases are linked to occupational contact with pigs and are usually caused by serotype 2 sequence type 1 (ST1) and ST20 strains, whereas other serotype 2 genotypes seldom cause human disease on the continent.

**Case Summary:**

A 55-year-old male pig farmer from Brittany, France, presented with acute confusion, fever, and nuchal rigidity consistent with meningitis. Cerebrospinal fluid cultures were negative, but blood cultures grew *S. suis*. Empirical treatment with cefotaxime and dexamethasone was initiated, followed by high-dose amoxicillin once antimicrobial susceptibility results confirmed β-lactam sensitivity. The patient required 5 days of intensive care and 7 additional days in a general ward and then completed 8 days of home intravenous ceftriaxone, fully recovering without hearing loss, a frequent sequela of *S. suis* meningitis. The isolate was typed as serotype 2. It belonged to the ST25 lineage and was genetically closely related to North American strains.

**Conclusion:**

This case serves as a reminder that *S. suis* should be considered in meningitis among swine-exposed individuals and expands the known range of ST25 human infections to Western Europe, providing useful insights for regional surveillance efforts.

## INTRODUCTION

*Streptococcus suis is* a swine pathogen and zoonotic agent that most commonly presents in humans as meningitis, sepsis, or toxic shock-like syndromes (1, 2), and is strongly associated with exposure to pigs or raw pork products through occupational activities, and in Southeast Asia, the consumption of raw or undercooked pork (1–3). Although human *S. suis* disease is globally distributed, its burden varies markedly by region. In Thailand, Vietnam, and China, the organism is a major agent of adult bacterial meningitis, whereas infections remain infrequent in Africa, the Americas, and Australia (3–9). Europe represents approximately 10% of global human *S. suis* infections (10), which are primarily caused by highly pathogenic serotype 2 sequence type (ST) 1 strains (2, 10). The serotype 2 ST25 lineage, known to cause human disease in Southeast Asia and North America (3, 8), has not been documented in Western Europe. Here, we report a meningitis case in a French pig farmer caused by an isolate of this lineage, which we contextualize genomically within the ST25 population structure.

## CASE PRESENTATION

**Fig 1:**
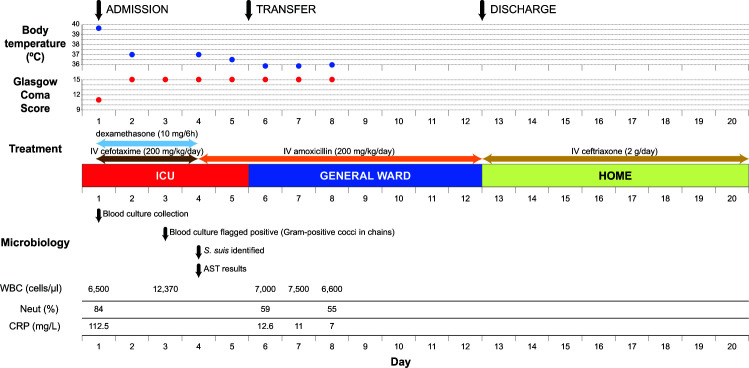
Clinical course, treatment timeline, and microbiologic findings for the patient with *S. suis* meningitis. From the top, the chart shows the dates of admission, transfer from ICU to general ward and discharge, changes in body temperature, Glasgow coma scores, treatment, dates of blood culture collection, microbiological identification of the causative agent and antimicrobial susceptibility testing, and changes in white blood cell counts, percentage of neutrophils, and level of C-reactive protein when available. Abbreviations: ICU, intensive care unit; IV, intravenous; AST, antimicrobial susceptibility testing; WBC, white blood cells; Neut, neutrophils; CRP, C-reactive protein.

A 55-year-old male pig farmer from Brittany, France, with no prior medical history, presented to the emergency department in February 2022 after being found unresponsive on his farm. His family reported fever and myalgia for 2 days. On admission, vital signs were blood pressure 150/80 mmHg, heart rate 80 bpm, respiratory rate 30 breaths/min, and body temperature 39.6°C. He was confused, with a Glasgow Coma Scale score of 11—eye opening 4 (spontaneous), verbal response 3 (inappropriate words), motor response 4 (withdrawal from pain) (Fig. 1). Physical examination revealed no rashes or petechiae or signs of trauma or external injuries, and nuchal rigidity was present. Pupillary reactions were normal, with no focal neurological deficits. Cardiovascular and respiratory examinations were unremarkable. Empirical intravenous (IV) antibiotic therapy with cefotaxime (200 mg/kg/day [12 g/day]) was initiated to cover common bacterial causes of meningitis such as *Streptococcus pneumoniae* and *Neisseria meningitidis*, along with intravenous (IV) dexamethasone (10 mg/6 h) to reduce inflammation (Fig. 1).

Initial blood work showed a white blood cell (WBC) count of 6,500 cells/mm^3^ (normal 4,000–10,000 cells/mm^3^) with 84% neutrophils (normal 40%–75%) and thrombocytopenia (platelet count 108,000 cells/mm^3^; normal 150,000–400,000 cells/mm^3^) (Fig. 1). C-reactive protein was elevated (112.5 mg/L; normal <5 mg/L), as was procalcitonin (0.54 µg/L; normal <0.05 µg/L). Blood glucose, renal and liver function were normal. No plasma alcohol was detected. Lumbar puncture was performed at admission prior to initiation of antimicrobial therapy. It yielded clear cerebrospinal fluid (CSF); analysis showed elevated protein (0.91 g/L, normal 0.15–0.45 g/L), glucose (3.8 mmol/L; normal 2.5–4.5 mmol/L), and 810 cells/mm^3^ (normal <5 cells/mm^3^) with 95% neutrophils, consistent with purulent meningitis. CSF Gram stain was negative, and cultures remained negative after 3 days. No molecular diagnostic testing was performed on CSF. Two sets of blood cultures collected at admission were processed using an automated blood culture system (BacT/ALERT, bioMérieux, Marcy-l’Étoile, France). Bottles were incubated until flagged positive, at which point Gram staining revealed Gram-positive cocci in chains. Subculture was performed on routine media. *S. suis* was identified by matrix-assisted laser desorption/ionization time-of-flight (MALDI-TOF) mass spectrometry (11) using a Microflex LT/SH mass spectrometer (Bruker Daltonics, Bremen, Germany). Spectra were compared with the Bruker Biotyper RUO database, yielding a high-confidence species identification (score ≥2.0). Antimicrobial susceptibility testing (AST) was performed using the VITEK 2 system (bioMérieux, Marcy-l’Étoile, France) with the AST-ST card. Results were interpreted according to EUCAST criteria. The isolate (NSUI0719) was susceptible to penicillin, amoxicillin, cefotaxime, ceftriaxone, and vancomycin and resistant to erythromycin and tetracycline. Treatment was adjusted to high-dose IV amoxicillin (200 mg/kg/day).

The patient remained in the intensive care unit for 5 days and improved rapidly. Computed tomography (CT) of the brain showed no edema, hemorrhage, mass effect, or hydrocephalus. Whole-body CT scan was normal, and transthoracic echocardiography revealed no signs of endocarditis. His temperature decreased (Fig. 1), and by day 5, he was alert, following commands, and had a Glasgow Coma Scale score of 15. WBC were normal and C-reactive protein decreased to 12.6 mg/L by day 6. The patient was transferred to a general ward, with continued amoxicillin until discharge, followed by intravenous ceftriaxone (2 g/day) from days 13 to 20 administered at home to facilitate outpatient parenteral antimicrobial therapy, given its once-daily dosing convenience (Fig. 1). Follow-up confirmed complete recovery without relapses or hearing loss.

**Fig 2:**
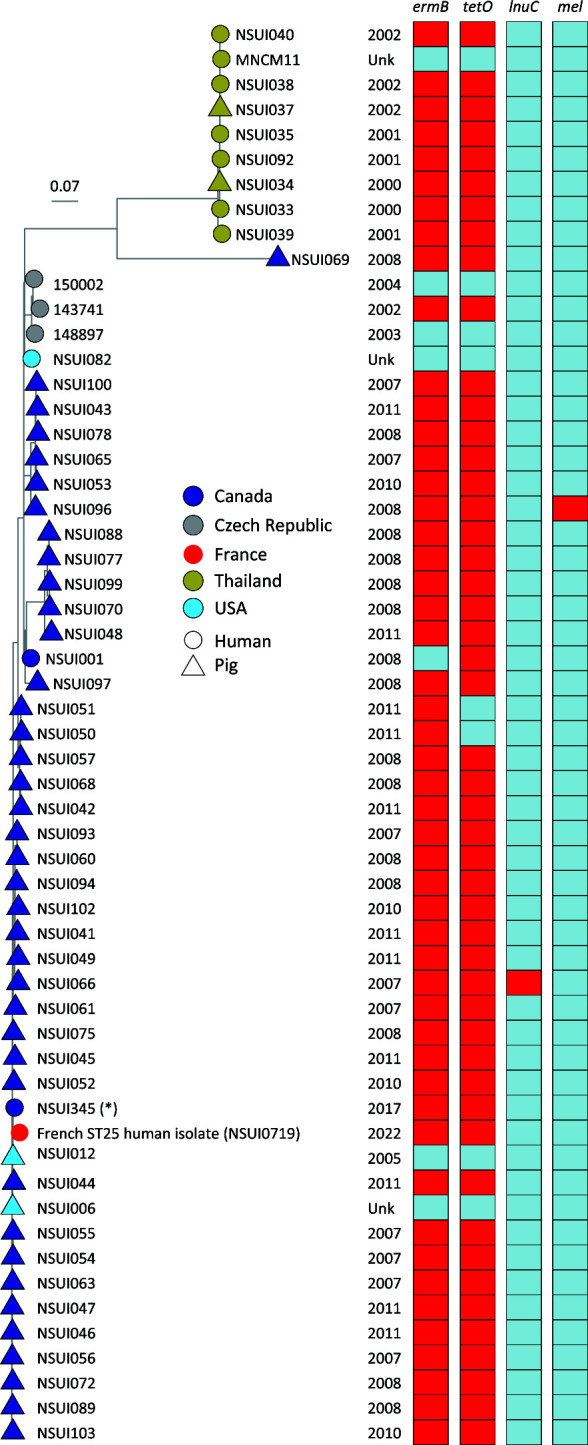
Phylogeny of *S. suis* ST25 isolates from human (circles) and swine (triangles) sources, with antimicrobial resistance (AMR) gene content indicated. Isolates are color-coded by country of origin. The French patient isolate (NSUI0719) clusters with Canadian strains, and the closest relative is an isolate recovered from a human case in Ontario, Canada (indicated by a star). ST25 isolates from the Czech Republic and Thailand form distinct subclades. The genetic relationships between strains were inferred, and AMR gene identified, using previously described bioinformatics approaches (14). AMR genes identified in the French isolate (*ermB* and *tetO*) were in agreement with the observed phenotypic resistance. Scale bar indicates nucleotide substitutions per site.

The co-agglutination test (12) assigned NSUI0719 to serotype 2. Whole genome sequencing was performed using an Illumina MiSeq instrument (Illumina, San Diego, Calif). Serotype was confirmed and ST determined using a validated bioinformatics workflow for *S. suis* (13). The isolate was classified as ST25. It was devoid of the virulence markers muramidase-released protein, extracellular factor, and suilysin (genes *mrp*, *epf,* and *sly*), typical of highly pathogenic ST1 strains (2). Phylogenetic analysis, performed as previously described (14), permitted comparison of NSUI0719 with all ST25 genome sequences available at public repositories, i.e., 3 human-derived ST25 strains from the Czech Republic (10), 39 swine- and 2 human-derived ST25 isolates from Canada, 2 swine- and 1 human-derived ST25 isolates from the United States, and 7 human- and 2 pig-derived ST25 isolates from Thailand (15). NSUI0719 was closely related to an ST25 strain from a human case in Ontario (8) and to ST25 isolates from diseased swine in Canada (15) but was distant from Czech and Thai isolates (Fig. 2).

Investigation of farm practices revealed that the patient had frequent direct contact with pigs and carcasses without consistent use of personal protective equipment. At the time of infection, no active *S. suis* disease was present on his farrow-only farm, which houses sows and suckling piglets, with weaning and off-site transfer at 28–35 days. Serotype 2 infections in post-weaning piglets at recipient farms were documented only until 2017. An isolate recovered in 2017 from a diseased piglet at a recipient farm (NSUI00716) was used to produce an autogenous vaccine administered to sows on the patient’s farm until late 2019. To assess a potential link, we sequenced its genome; bioinformatic typing assigned it to ST1, indicating it was unrelated to the isolate responsible for the patient’s infection. The patient reported no recent importation of pigs or pig products from countries known to harbor ST25 strains, including Canada, and no travel during the relevant period.

## DISCUSSION

*S. suis* infection can lead to meningitis, sepsis, and complications such as hearing loss (16–18). Early recognition and prompt antibiotic therapy are crucial. Initial management followed French national guidelines for adult community-acquired bacterial meningitis (19), with prompt empiric third-generation cephalosporin and dexamethasone, followed by targeted therapy after identification of *S. suis* and availability of susceptibility results. The 20-day antimicrobial course is consistent with reported management of *S. suis* meningitis, where CSF Gram stain and cultures are frequently negative (16). Clinicians should consider *S. suis* in the differential diagnosis of meningitis, particularly in patients with occupational exposure to pigs (e.g., farmers, abattoir workers, butchers) or recreational exposure to wild boars (1, 2, 20). Alternative transmission routes, such as the consumption of raw or undercooked pork, have been described (3, 9) but were not reported in this case and are uncommon in Western Europe.

Although human *S. suis* infections are less frequent in Europe than in parts of Asia, a retrospective survey identified 236 previously unrecognized cases between 1990 and 2022 (10). European cases have predominantly involved more virulent serotype 2 ST1 or ST20 strains (10), whereas ST25 infections have mainly been reported in North America and parts of Southeast Asia (3, 8). We, therefore, performed genomic analysis to place the isolate within the broader ST25 population structure. Although NSUI0719 was closely related to a human ST25 strain from Ontario, Canada, no direct links to Canada could be established.

Low-level circulation of ST25 serotype 2 *S. suis* in pigs in Western Europe could explain the origin of this isolate; however, available surveillance data do not support this hypothesis. Among 165 serotype 2 isolates recovered from diseased pigs in France between 2017 and 2022, nearly 70% were ST1, with the remainder comprising genotypes unrelated to ST25, which was not detected (14). The retrospective identification of ST25 among human infections in the Czech Republic (10) indicates its presence in Central Europe; however, ST25 was not detected in a large survey of 528 swine isolates from that country (21). These data underscore the limited understanding of the ecology of minority *S. suis* lineages capable of causing human disease. ST25 may have a broader geographic distribution than currently recognized, as recent human cases have been reported in China (22, 23), where ST25 is also rarely detected in swine (24). Broader genomic surveillance integrating human, swine, and environmental sampling will be needed to determine whether ST25 is truly emerging or underdetected in Europe.

### Conclusion

Clinically, this case reinforces that *S. suis* should remain in the differential diagnosis of meningitis among people with swine exposure. Epidemiologically, the first identification of an ST25 infection in Western Europe expands the known range of this lineage and highlights gaps in surveillance that obscure its reservoirs, distribution, and zoonotic potential.

## Data Availability

Genome data for the two isolates whose genomes were sequenced in this investigation (NSUI0719, the ST25 strain responsible for the human infection, and NSUI0716, the ST1 strain used in the autogenous vaccine) have been deposited in NCBI’s Sequence Read Archive under accession numbers SRR34985968 and PRJNA1462058, respectively. Accession numbers for comparator genomes are provided in previous publications (8, 13, 15), with the exception of strain MNCM11, whose genome can be retrieved from NCBI’s RefSeq using accession number GCF_016584805.1.
